# Resuscitation fluid composition affects hepatic inflammation in a murine model of early sepsis

**DOI:** 10.1186/s40635-017-0118-5

**Published:** 2017-01-19

**Authors:** Amanda L. Patrick, Peter M. Grin, Nicole Kraus, Michelle Gold, Matthew Berardocco, Patricia C. Liaw, Alison E. Fox-Robichaud, on behalf of the Canadian Critical Care Translational Biology Group

**Affiliations:** 1 Department of Medicine, McMaster University, Hamilton, Ontario Canada; 2 Department of Medical Sciences, McMaster University, Hamilton, Ontario Canada; 3 Thrombosis and Atherosclerosis Research Institute, McMaster University, DBRI C5-106, 237 Barton St. East, Hamilton, ON L8L 2X2 Canada

**Keywords:** Sepsis, Resuscitation fluid, Albumin, Leukocyte recruitment, Cytokine, Chemokine, Cell-free DNA, Cecal ligation and puncture, Intravital microscopy

## Abstract

**Background:**

Fluid resuscitation is a crucial therapy for sepsis, and the use of balanced fluids and/or isotonic albumin may improve patient survival. We have previously demonstrated that resuscitation with normal saline results in increased hepatic leukocyte recruitment in a murine model of sepsis. Given that clinical formulations of albumin are in saline, our objectives were to develop a novel balanced electrolyte solution specifically for sepsis and to determine if supplementing this solution with albumin would improve the inflammatory response in sepsis.

**Methods:**

We developed two novel buffered electrolyte solutions that contain different concentrations of acetate and gluconate, named Seplyte L and Seplyte H, and administered these solutions with or without 5% albumin. Normal saline with or without albumin and Ringer’s lactate served as controls. Sepsis was induced by cecal ligation and puncture (CLP), and the liver microvasculature was imaged in vivo at 6 h after CLP to quantify leukocyte recruitment. Hepatic cytokine expression and plasma cell-free DNA (cfDNA) concentrations were also measured.

**Results:**

Septic mice receiving either Seplyte fluid showed significant reductions in hepatic post-sinusoidal leukocyte rolling and adhesion compared to normal saline. Hepatic cytokine concentrations varied in response to different concentrations of acetate and gluconate in the novel resuscitation fluids but were unaffected by albumin. All Seplyte fluids significantly increased hepatic TNF-α levels at 6 h compared to control fluids. However, Seplyte H exhibited a similar cytokine profile to the control fluids for all other cytokines, whereas mice given Seplyte L had significantly elevated IL-6, IL-10, KC (CXCL1), and MCP-1 (CCL2). Plasma cfDNA was generally increased during sepsis, but resuscitation fluid composition did not significantly affect cfDNA concentrations.

**Conclusions:**

Electrolyte concentrations and buffer constituents of resuscitation fluids can modulate hepatic cytokine production and leukocyte recruitment in septic mice, while the effects of albumin are modest during early sepsis. Therefore, crystalloid fluid choice should be an important consideration for resuscitation in sepsis, and the effects of fluid composition on inflammation in other organ systems should be studied to better understand the physiological impact of this vital sepsis therapy.

**Electronic supplementary material:**

The online version of this article (doi:10.1186/s40635-017-0118-5) contains supplementary material, which is available to authorized users.

## Background

Sepsis is characterized by severe inflammation along with organ dysfunction resulting from a dysregulated host response to infection and requires timely fluid resuscitation to improve patient survival. The main treatment regimen for patients with sepsis involves restoring microvascular blood flow using intravenous fluid resuscitation within 6 h of patient presentation, identifying and eliminating the source of infection by administering appropriate antimicrobials, and providing supportive care for organ dysfunction [1]. Although fluid resuscitation is a key component of sepsis treatment, the ideal choice of intravenous fluid for early resuscitation of septic patients remains controversial [2, 3].

Traditional crystalloid fluids, such as normal saline and Ringer’s lactate, remain as the most commonly used fluids by intensivists to manage sepsis and early septic shock [4, 5]. Colloid fluids, consisting of proteins or starches suspended in electrolyte solutions, have also been used for resuscitation of patients with sepsis. With evidence that colloid fluids containing hydroxyethyl starches increase the risk of acute kidney injury and mortality in critically ill patients [6, 7], and uncertainty about the safety of gelatins [8], clinicians are now limited to albumin. The Surviving Sepsis Campaign guidelines currently recommend crystalloids as first-line therapy and colloid solutions containing albumin when excessive fluid resuscitation is required [1]. While current evidence suggests that resuscitation with isotonic albumin does not cause harm, the question of benefit remains contested [9–13]. Albumin is typically suspended in saline-based solutions containing 109–137 mM concentrations of chloride [Bram Rochwerg, personal communication], which may potentiate hyperchloremic acidosis. Indeed, normal saline has been linked to hyperchloremic metabolic acidosis during large-volume resuscitation [14]. The fact that commonly used resuscitation fluids may have serious adverse effects justifies the need to investigate how traditional fluids compare to emerging balanced fluids, electrolyte solutions containing ion and buffer concentrations that closely resemble those of healthy plasma [15–19]. Current evidence suggests that resuscitation with balanced fluids may reduce the risk of mortality in patients with sepsis [11, 20], but the physiological mechanisms involved are unclear.

It is clear that all resuscitation fluids are not equal and an optimized fluid is needed for use in sepsis treatment. We previously demonstrated that resuscitation fluid composition has dramatic effects on hepatic leukocyte recruitment in an animal model of early sepsis [21, 22]. Since the liver is central to the inflammatory response in sepsis, and hepatic dysfunction has been associated with long-term mortality from septic shock [23], we focused on assessing the inflammatory state of the hepatic microvasculature in response to various crystalloids, colloids, and balanced resuscitation fluids during sepsis. To accomplish this, we developed two novel balanced fluids that contain ion concentrations similar to plasma and are buffered with gluconate in addition to either lower or higher concentrations of acetate, named Seplyte L or H, respectively. We tested these novel solutions with and without the addition of albumin and compared them to traditional fluids in a mouse model of early sepsis induced by cecal ligation and puncture (CLP). The inflammatory response was assessed using intravital microscopy of the liver microcirculation and hepatic cytokine concentrations, along with measurement of plasma cell-free DNA (cfDNA)—a biomarker that indicates poor prognosis when elevated in sepsis [24, 25]. We hypothesized that fluid resuscitation with the novel Seplyte fluids would reduce the hepatic inflammatory response during early sepsis and that supplementing these fluids with albumin would enhance this reduction.

## Methods

### Novel buffered electrolyte solutions

Seplyte L and Seplyte H solutions were prepared in 1 L of Millipore water with the compounds and concentrations listed in Table 1. Once dissolved and pH adjusted to 7, the solutions were filter sterilized using Millipore Stericup and Steritop Express Plus 0.22 μm filters.

**Table 1:** Constituents of novel balanced crystalloid solutions

Compound	Mass (g/L)	Ion	Concentration (mmol/L)
A) Seplyte L (low acetate, no potassium)			
MgCl_2_	0.119	Na^+^	142
CaCl_2_	0.277	Cl^−^	103
NaCl	5.581	Mg^2+^	1.25
NaC_6_H_11_O_7_ (sodium gluconate)	4.035	C_6_H_11_O_7_^−^	18.5
NaCH_3_CO_2_ (sodium acetate)	2.297	CH_3_COO^−^	28
		Ca^2+^	2.5
B) Seplyte H (high acetate, no potassium)			
MgCl_2_	0.119	Na^+^	142
CaCl_2_	0.277	Cl^−^	103
NaCl	5.611	Mg^2+^	1.25
NaC_6_H_11_O_7_ (sodium gluconate)	2.618	C_6_H_11_O_7_^−^	12
NaCH_3_CO_2_ (sodium acetate)	2.789	CH_3_COO^−^	34
		Ca^2+^	2.5

A stock solution of sodium acetate and sodium gluconate was prepared at 10 mg/mL with the ratio of molecular weights of each ion in the compound taken into account. A 10 mg/mL stock solution of sodium sulfate was prepared for use as the internal standard. Calibration standards were prepared in the range of 0.6–50 μg/mL, with threefold dilutions between each standard. Samples of Seplyte L and Seplyte H were diluted by a factor of 100 for optimal detection. Each calibration standard and samples contained a constant concentration of internal standard at 10 μg/mL.

Electrospray ionization mass spectrometry was used to examine the stability of the novel electrolyte solutions by examining the concentrations of acetate and gluconate ions over time. The Micromass Quattro Ultima LC-ESI/APCI Triple Quadrupole Mass Spectrometer was used for all measurements. Injections of 10 μL were made using a syringe into the six-port injector equipped with a 10-μL sample loop. The sample was then carried into the ionization chamber of the mass spectrometer by the mobile phase (water/methanol, 50:50) at 50 μL/min. The instrument was set to a source temperature of 800 °C and dissolution temperature of 2100 °C, with cone gas flowing at approximately 130 L/h and dissolution gas at 900–1200 L/h. All analyses were made in negative ion mode, with a capillary voltage of 3.00 kV applied at the tip and cone voltage set to 35 V. Blanks were injected between samples to minimize carry-over. Selected ion monitoring was chosen as the scanning method, and smoothing algorithms were applied to the data to allow the area under the curve to be integrated, reflecting the strength of the signal. The responses of the target analytes were measured in terms of the internal standard response. The equation of the calibration curve was applied to the relative values to determine the concentration of the acetate and gluconate ions in solution. Concentration analyses were performed for one batch at approximately 3 months after the date of preparation and another batch at approximately 1 year. Data is reported for the 1-year analysis (Table 2). Various storage conditions were examined to determine long-term stability when stored in plastic or glass and under light or darkness.

**Table 2:** Concentrations of gluconate and acetate in novel Seplyte solutions stored under various conditions, as measured by mass spectroscopy

Solution	Storage condition	Gluconate (μg/mL)	Acetate (μg/mL)
Seplyte L Expected: 40.35 μg/mL gluconate 22.97 μg/mL acetate	Prepared fresh	38.64	12.02
	Plastic light exposed	36.64	12.80
	Plastic dark	40.04	14.64
	Glass light	37.19	14.43
	Glass dark	40.00	14.89
Seplyte H Expected: 26.18 μg/mL gluconate 27.89 μg/mL acetate	Prepared fresh	23.12	15.35
	Plastic light exposed	112.48^a^	19.66
	Plastic dark	26.15	17.56
	Glass light	25.48	18.92
	Glass dark	27.14	19.29

^a^Markedly different from expected concentration

### Animals

Wild-type, *Helicobacter hepaticus-*negative, C57BL/6 male mice were purchased from Taconic Laboratories (Georgetown, NY, USA) for the experiments. All mice were housed under pathogen-free conditions and had access to standard rodent food and water ad libitum. Experiments were performed on mice between 7 and 10 weeks of age. This study received approval by the Animal Research Ethics Board at McMaster University and met all criteria outlined by the Canadian Council on Animal Care.

### Preliminary fluid studies in endotoxemia

The acute hepatic response to normal saline, Ringer’s lactate, and the two novel Seplyte solutions was assessed through intravital microscopy of the mouse liver at 4 h after injection of lipopolysaccharide (LPS) or control (see Additional file 1).

### Cecal ligation and puncture

Polymicrobial sepsis was induced by CLP as previously described by Baker et al. [26], with the following modifications: all animals received subcutaneous analgesic (buprenorphine; 0.05 mg/kg) and underwent laparotomy while under isoflurane anesthesia (Baxter, Mississauga, ON, Canada). Using sterile technique, an intravenous line (polyethylene 10 mm, VWR Canada, Mississauga, ON, Canada) was introduced by cutdown into the right internal jugular vein and tunneled subcutaneously to the back of the neck. A 1-cm portion of the distal cecum was then exteriorized, ligated, and punctured once with an 18-gauge needle, and a small amount of fecal matter was extruded. The cecum was then replaced into the abdominal cavity, and the incision was sutured to close the muscle and skin independently. Sham-operated mice were treated identically except for the ligation and puncture of the cecum. All animals were resuscitated according to the experimental design with an injection through the jugular vein catheter. The catheter was then flushed with 20 μL of heparinized saline to prevent blood from clotting and occluding the catheter.

### Experimental design

Sham and CLP animals were randomized to receive one of the following resuscitation fluids: normal saline, Ringer’s lactate, normal saline with 5% albumin (*w*/*v*; Baxter), Seplyte L, Seplyte L with 5% albumin, Seplyte H, or Seplyte H with 5% albumin. Solutions containing 5% albumin were prepared using endotoxin-free albumin from Amsbio (Lake Forrest, CA, USA). Fluids were administered intravenously immediately following surgery and 4 h later. The crystalloid solutions (without albumin) were given as 0.5-mL boluses at each time point, based on the dosing of 20 mL/kg used in clinical practice. The colloid solutions (containing albumin) were given as 0.125-mL injections. The ratio of 1:4 colloid to crystalloid is based on the oncotic properties of these solutions [27].

### Intravital microscopy

The hepatic microcirculation was examined using trans-illumination intravital microscopy 6 h after inducing sepsis, as previously described [21]. All mice were anesthetized with an intraperitoneal injection of ketamine (200 mg/kg) and xylazine (10 mg/kg). The abdomen was re-opened with a midline incision extending near the costal margin. Mice were then placed in a left lateral position on a Plexiglas microscope stage with the left lobe of the liver exteriorized and covered with plastic wrap (Saran Wrap) in order to prevent dehydration and movement from respiration. Body temperatures were maintained by the use of an infrared heat lamp and a heated water-circulating jacket built into the stage.

Hepatic sinusoids and post-sinusoidal venules were visualized by intravital microscopy (Zeiss Inverted Axiovert S100, Toronto, ON, Canada). Blood flow through the microcirculation was captured with an attached camera (Newvicon DAGE- MTI, Michigan City, IN, USA) and viewed on a monitor (Panasonic CT-2086YD, Mississauga, ON, Canada). The images were recorded using a DVD recorder (Panasonic DMR-EH55) for offline analysis. Within each field of view, one post-sinusoidal venule or approximately 6–10 sinusoids were observed. The number of adherent and rolling leukocytes was quantified during video playback analysis. Leukocytes were considered rolling if tethering to a given venule with a torsional motion was observed. Adherent leukocytes became immobilized along the endothelial wall and remained stationary for a minimum of 30 s. Vessel images were recorded at time 0 and 10 min later, for the duration of 2 min at each time point, to obtain an average of leukocytes per minute per field of view (160 × 200 μm).

### Sample collection

Following intravital microscopy, animals were sacrificed via cardiac puncture followed by cervical dislocation. Blood was collected from the left ventricle with a sodium-citrated syringe and processed within 30 min. The blood was centrifuged at 1500×*g* and the plasma portion stored at −80 °C for further cfDNA analysis. Remaining whole blood was smeared onto a slide for leukocyte differential counts using a compound light microscope. Slides were stained for differential leukocyte identification using a Hemacolor Staining Set (EMD Millipore, Gibbstown, NJ, USA). The leukocyte differential values were based on counting 100 cells per slide to determine the percentage of neutrophils, lymphocytes, monocytes, and eosinophils. Whole blood was also mixed with 3% acetic acid and 1% crystal violet dye (5:44:1, blood/acetic acid/dye ratio) to determine total leukocyte counts using a hemocytometer. Absolute leukocyte differentials were calculated by multiplying the proportion of each type of leukocyte by the total leukocyte count. Lastly, tissue samples of the liver were collected, snap frozen in liquid nitrogen, and stored at −80 °C for cytokine assays.

### Quantification of plasma cell-free DNA

For isolation of cfDNA from mouse plasma, the Qiagen QIAamp DNA Mini Blood Mini Kit was used according to the manufacturer’s instructions (Qiagen, Valencia, CA, USA) after spinning plasma at 16,800×*g* for 10 min. A spectrophotometer (Eppendorf BioPhotometer Plus, Hamburg, Germany) was used to quantify levels of cfDNA. The optical density (OD) reflects the amount of DNA present and was measured at excitation and emission wavelengths of 260 and 280 nm, respectively. The OD/260 nm values were converted to nanogram per microliter using the conversion of 1 OD/260 nm = 50 ng/μL DNA. A standard calibration curve was obtained using known ssDNA concentrations (UltraPure Salmon Sperm DNA, ThermoFisher Scientific, Burlington, ON, Canada) ranging from 0 to 10 mg/mL and was used to confirm the accuracy of cfDNA concentrations calculated using the OD method.

### Cytokine profiling

A Bio-Plex Cell Lysis Kit (Bio-Rad Laboratories, Mississauga, ON, Canada) was used to prepare liver samples for cytokine assays according to the manufacturer’s instructions. Homogenized samples were centrifuged at 4500×*g* for 10 min, and the supernatant was collected and frozen at −80 °C. A custom Mouse 7-Plex Cytokine Assay (Bio-Plex Pro Assay) was designed to measure IL-1β, IL-6, IL-10, IL-17, TNF-α, MCP-1 (CCL2), and KC (CXCL1) in liver tissue lysates. Homogenized liver supernatant was prepared according to instructions, and samples were analyzed on a Bio-Plex Luminex 200 System at 635- and 532-nm wavelengths using Bio-Plex Manager 6.0 software. To determine the concentration of each analyte per milligram of total hepatic protein, the mass of each analyte measured through the Bio-Plex assay was divided by the corresponding Bradford Protein Assay value for the sample.

### Statistical analyses

Statistical analyses were performed using GraphPad Prism 5.0 (La Jolla, CA, USA). Data are presented as mean ± standard error of the mean (SEM). Data obtained from multiple groups were analyzed using one-way analysis of variance (ANOVA) followed by Bonferroni post hoc tests. Differences between groups were considered to be statistically significant at *p* < 0.05.

## Results

### Novel resuscitation fluids

Both of the novel Seplyte fluids remained stable under a variety of storage conditions over the course of 1 year, except for Seplyte H stored in plastic and exposed to light (Table 2).

### Hepatic leukocyte recruitment in response to resuscitation with novel Seplyte fluids

**Fig. 1:**
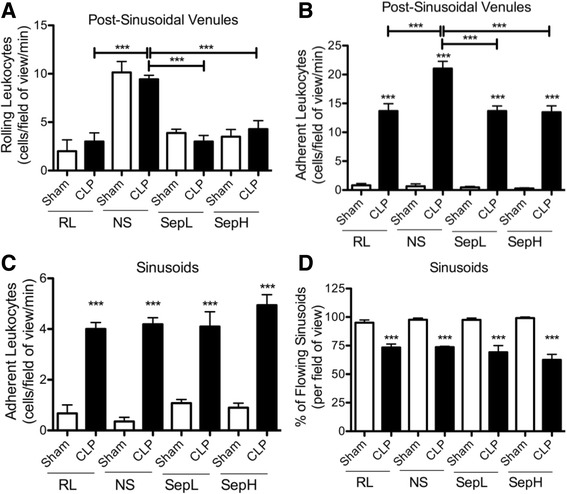
Leukocyte recruitment in the hepatic microcirculation in response to resuscitation with novel or traditional crystalloids. Intravital microscopy was used to measure leukocyte rolling (**a**) and adhesion (**b**) in hepatic post-sinusoidal venules, as well as sinusoidal leukocyte adhesion (**c**) and blood flow (**d**) in CLP or sham mice at 6 h after surgery. All mice were resuscitated with either 20 mL/kg of Ringer’s lactate, normal saline, or novel Seplyte L or H fluids, in accordance with clinical dosing practice. Fluids were administered intravenously immediately following surgery and 4 h later. Data are presented as mean ± SEM with *n* = 6–8 for all groups. ***p < 0.001; *asterisk directly above error bar* indicates comparison to sham control. *CLP* cecal ligation and puncture, *RL* Ringer’s lactate, *NS* normal saline, *SepL* Seplyte L, *SepH* Seplyte H

To compare the effects of traditional crystalloids and novel balanced Seplyte fluids on leukocyte recruitment in vivo, we examined the hepatic microcirculation using intravital microscopy. Leukocyte rolling in post-sinusoidal venules of mice undergoing CLP was similar to that observed in corresponding sham groups (Fig. 1a). However, normal saline resuscitation significantly increased post-sinusoidal leukocyte rolling compared to all other fluid treatments. Leukocyte adhesion in hepatic post-sinusoidal venules was significantly elevated in septic mice relative to shams regardless of which traditional or balanced crystalloid was administered (Fig. 1b). Furthermore, septic mice receiving normal saline experienced significantly more post-sinusoidal adhesion than septic mice receiving any other crystalloid fluid. Mice subjected to CLP also had significantly increased sinusoidal leukocyte adhesion compared to sham controls for each crystalloid fluid, although there were no significant differences between fluids (Fig. 1c). Sinusoidal blood flow was significantly decreased in septic mice compared to shams for all crystalloid fluid treatments but also did not significantly differ between fluids (Fig. 1d). Taken together, these results demonstrate that the novel Seplyte fluids have similar effects to those of Ringer’s lactate on leukocyte recruitment in the liver during sepsis. On the other hand, leukocyte recruitment in the liver is increased in response to normal saline resuscitation.

### Crystalloid fluid composition affects hepatic cytokine production during sepsis

**Fig. 2:**
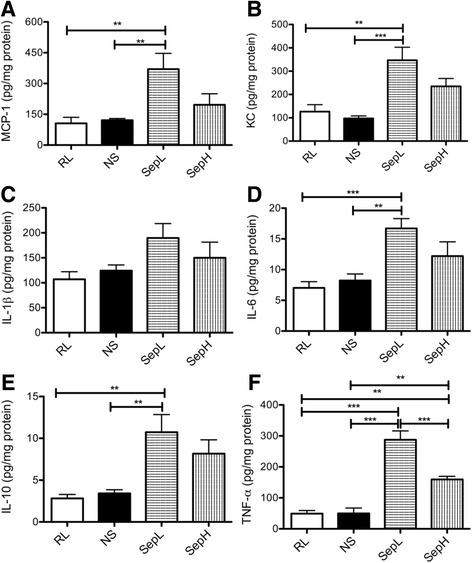
Crystalloid fluid composition influences hepatic cytokine production by septic mice. Concentrations of MCP-1 (**a**), KC (**b**), IL-1β (**c**), IL-6 (**d**), IL-10 (**e**), and TNF-α (**f**) were measured in mouse livers at 6 h after CLP. Data are presented as mean ± SEM with *n* = 5–6 for each group. **p < 0.01, ***p < 0.001. *RL* Ringer’s lactate, *NS* normal saline, *SepL* Seplyte L, *SepH* Seplyte H

In order to further assess the hepatic inflammatory response to the novel balanced crystalloid fluids during sepsis, we analyzed hepatic cytokine production in mice subjected to either CLP or sham surgery. Septic mice generally had significantly greater hepatic cytokine expression compared to sham mice for each resuscitation fluid, but at this time point, IL-17 was only present in trace amounts (<1 pg/mg hepatic protein) in all sham and CLP groups (data not shown). Septic mice resuscitated with Seplyte H produced similar concentrations of hepatic cytokines as mice treated with control fluids (Fig. 2a–e), with the exception of TNF-α (Fig. 2f). Interestingly, both Seplyte fluids significantly increased TNF-α compared to control fluids, with Seplyte L resulting in significantly greater TNF-α concentrations than Seplyte H (Fig. 2f). Furthermore, septic mice receiving Seplyte L had the highest hepatic concentrations of all measured cytokines, and these concentrations were significantly different from both control fluids for all cytokines except IL-1β (Fig. 2a–f). Since Seplyte L and Seplyte H have variable acetate and gluconate concentrations in the presence of identical concentrations of all other constituents, these findings suggest that acetate and gluconate can affect hepatic cytokine production, especially that of TNF-α. Collectively, these results demonstrate that crystalloid fluid composition can have a dramatic impact on inflammatory cytokine production in the liver during early sepsis.

### Hepatic leukocyte recruitment and cytokine production in response to fluid resuscitation with albumin

**Fig. 3:**
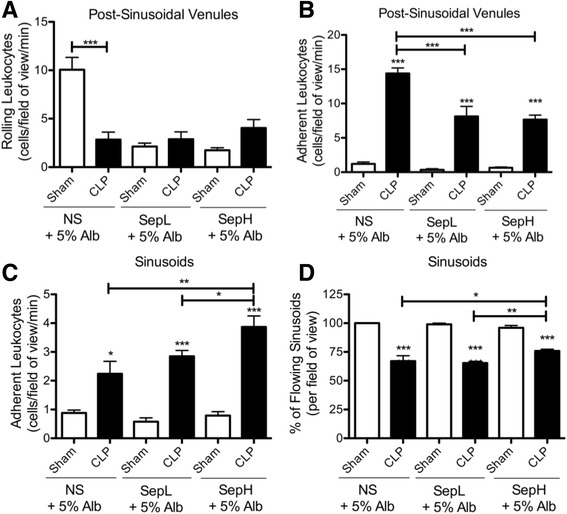
Leukocyte recruitment in the hepatic microcirculation in response to resuscitation with colloid solutions of albumin. Intravital microscopy was used to measure leukocyte rolling (**a**) and adhesion (**b**) in hepatic post-sinusoidal venules, as well as sinusoidal leukocyte adhesion (**c**) and blood flow (**d**) in CLP or sham mice at 6 h after surgery. All mice were resuscitated with either 5 mL/kg of normal saline containing 5% albumin, or novel Seplyte L or H fluids containing 5% albumin, in accordance with clinical dosing practice. Fluids were administered intravenously immediately following surgery and 4 h later. Data are presented as mean ± SEM with *n* = 5–6 for each group. *p < 0.05, **p < 0.01, ***p < 0.001; *asterisk directly above error bar* indicates comparison to sham control. *CLP* cecal ligation and puncture, *NS* normal saline, *SepL* Seplyte L, *SepH* Seplyte H, *5% Alb* 5% albumin

To determine whether addition of albumin to the novel Seplyte fluids could impact inflammation in the liver, we first used intravital microscopy to quantify hepatic leukocyte recruitment in vivo. Leukocyte rolling in the hepatic post-sinusoidal venules was similar between sham and CLP groups resuscitated with either Seplyte L or H + 5% albumin, whereas septic mice treated with normal saline + 5% albumin paradoxically had significantly decreased rolling compared to sham controls (Fig. 3a). On the contrary, CLP-induced sepsis resulted in significant increases in post-sinusoidal leukocyte adhesion relative to sham controls for all fluid treatment groups (Fig. 3b). Furthermore, septic mice resuscitated with either Seplyte L or H containing albumin had significantly reduced post-sinusoidal leukocyte adhesion compared to mice treated with normal saline containing albumin. Similar to hepatic post-sinusoidal venules, leukocyte adhesion in hepatic sinusoids was significantly increased in response to CLP in all fluid treatment groups (Fig. 3c). However, hepatic sinusoidal adhesion was greatest in mice treated with Seplyte H + 5% albumin. Interestingly, despite increases in sinusoidal adhesion which tend to obstruct blood flow, septic mice treated with Seplyte H + 5% albumin also had the greatest percentage of flowing sinusoids when compared to either normal saline or Seplyte L with albumin (Fig. 3d). Since sinusoidal blood flow was significantly decreased in response to sepsis for all resuscitation fluids, these findings suggest that the osmotic effects of albumin are not sufficient to restore sinusoidal blood flow in septic mice. Overall, the patterns of hepatic leukocyte recruitment in response to fluid resuscitation with albumin appear similar to those observed in response to the crystalloid fluids alone (Figs. 1 and 3), though resuscitating with albumin appears to modestly ameliorate the increases in post-sinusoidal leukocyte adhesion during sepsis (compare Figs. 1b and 3b). Taken together, these observations suggest that supplementing resuscitation fluids with albumin has a modest reduction on leukocyte recruitment in the hepatic microcirculation.

**Fig. 4:**
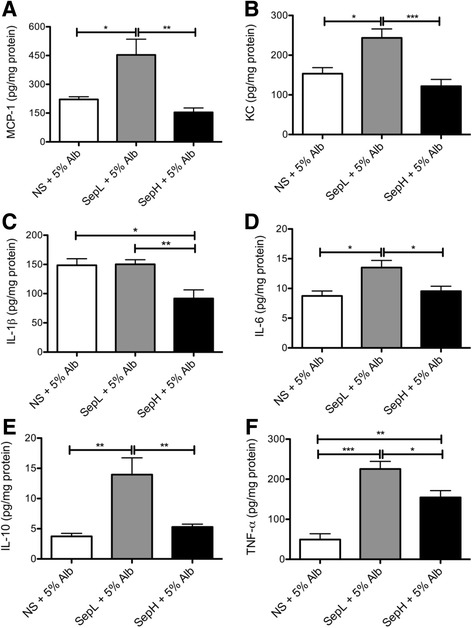
Hepatic cytokine production by septic mice in response to colloid resuscitation fluids. Concentrations of MCP-1 (**a**), KC (**b**), IL-1β (**c**), IL-6 (**d**), IL-10 (**e**), and TNF-α (**f**) were measured in mouse livers at 6 h after CLP. Data are presented as mean ± SEM with *n* = 6 for all groups. *p < 0.05, **p < 0.01, ***p < 0.001. *NS* normal saline, *SepL* Seplyte L, *SepH* Seplyte H, *5% Alb* 5% albumin

To further assess any effects of albumin on hepatic inflammation, we measured hepatic concentrations of various pro- and anti-inflammatory cytokines relevant in sepsis. Similar to our findings in the crystalloid fluids alone (Fig. 2), we observed significant increases in MCP-1, KC, IL-6, IL-10, and TNF-α, but not IL-1β, in septic mice resuscitated with Seplyte L + 5% albumin when compared to normal saline + 5% albumin (Fig. 4a–f). Interestingly, TNF-α remained significantly elevated for both Seplyte fluids supplemented with albumin in comparison to normal saline with albumin (Fig. 4f), with similar concentrations as observed for the crystalloid fluids alone (Fig. 2f). The only notable effect of albumin on the hepatic cytokine profile that we observed in septic mice was a significant decrease in IL-1β in response to Seplyte H + 5% albumin (Fig. 4c), which differed from the trends observed for crystalloid fluids alone (Fig. 2c). Therefore, these data suggest that albumin has a modest effect on hepatic cytokine concentrations only when incorporated into the novel Seplyte H fluid.

### cfDNA levels

**Fig. 5:**
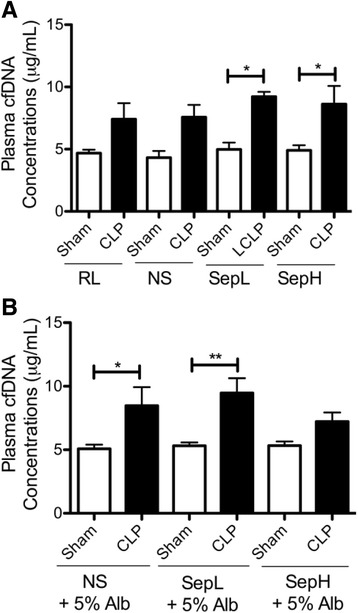
Cell-free DNA concentrations measured in septic mice or sham controls resuscitated with either crystalloid (**a**) or colloid fluids (**b**). Data are presented as mean ± SEM with *n* = 5–7 for each group. *p < 0.05, **p<0.01. *CLP* cecal ligation and puncture, *RL* Ringer’s lactate, *NS* normal saline, *SepL* Seplyte L, *SepH* Seplyte H, *5% Alb* 5% albumin

For all resuscitation fluids, cfDNA values trended towards increases in CLP mice compared to the corresponding shams, and these comparisons were significantly different for Seplyte L with or without albumin, Seplyte H alone, and normal saline with albumin (Fig. 5a, b). Plasma cfDNA concentrations in septic mice did not significantly differ among the various crystalloid solutions. Furthermore, adding albumin to each crystalloid fluid did not significantly affect cfDNA concentrations, except for modestly decreasing cfDNA in septic mice treated with Seplyte H + 5% albumin. Taken together, these data suggest that cfDNA is generally elevated early in CLP-induced sepsis and its utility as a biomarker should not be affected by the use of different resuscitation fluids.

### Absolute circulating leukocyte counts

To assess the effects of resuscitation fluid composition on systemic leukocyte counts, we measured circulating levels of neutrophils, lymphocytes, and monocytes in sham and septic mice. Circulating neutrophil counts were significantly lower in septic mice for all fluid treatments, except Seplyte H with albumin, in comparison to shams (Table 3). Among septic mice, normal saline with albumin resulted in the highest circulating neutrophil counts, which were significantly greater than those found in response to Seplyte L with albumin. With respect to circulating lymphocytes, there were no significant differences between any treatment groups (Table 3). Absolute circulating monocyte counts were significantly lower in septic mice receiving Ringer’s Lactate, normal saline, or Seplyte L when compared to sham mice receiving the same fluids, although there were no significant differences between any two fluid treatments in septic mice (Table 3). Taken together, these findings suggest that resuscitation fluid composition has minimal to no effect on circulating leukocyte numbers during sepsis.

**Table 3:** Mean absolute systemic leukocyte counts (±SEM) in response to resuscitation fluid treatment in C57BL/6 mice undergoing sham or CLP surgery

Fluid type	Sham (×10^9^ cells/L)	CLP (×10^9^ cells/L)
A) Neutrophils		
Ringer’s lactate	1.22 ± 0.18	0.35 ± 0.04*
Normal saline	1.66 ± 0.15	0.39 ± 0.06*
Normal saline + 5% albumin	2.69 ± 0.17	0.90 ± 0.22*
Seplyte L	1.63 ± 0.11	0.46 ± 0.11*
Seplyte L + 5% albumin	1.83 ± 0.16	0.20 ± 0.05*^
Seplyte H	2.08 ± 0.15	0.68 ± 0.24*
Seplyte H + 5% albumin	1.08 ± 0.23	0.53 ± 0.14
B) Lymphocytes		
Ringer’s lactate	2.20 ± 0.18	1.60 ± 0.26
Normal saline	1.83 ± 0.17	1.76 ± 0.15
Normal saline + 5% albumin	1.61 ± 0.22	1.44 ± 0.16
Seplyte L	1.65 ± 0.08	1.92 ± 0.31
Seplyte L + 5% albumin	1.78 ± 0.16	2.56 ± 0.38
Seplyte H	1.49 ± 0.22	2.22 ± 0.38
Seplyte H + 5% albumin	1.08 ± 0.22	1.50 ± 0.21
C) Monocytes		
Ringer’s lactate	0.23 ± 0.03	0.07 ± 0.01*
Normal saline	0.27 ± 0.05	0.13 ± 0.03*
Normal saline + 5% albumin	0.19 ± 0.04	0.10 ± 0.03
Seplyte L	0.32 ± 0.06	0.16 ± 0.03*
Seplyte L + 5% albumin	0.28 ± 0.10	0.06 ± 0.26
Seplyte H	0.14 ± 0.05	0.15 ± 0.03
Seplyte H + 5% albumin	0.18 ± 0.06	0.05 ± 0.02

*p < 0.05 compared to sham, ^*p* < 0.05 compared to normal saline + 5% albumin CLP

## Discussion

In this study, we compared the effects of our novel balanced Seplyte solutions with clinically utilized fluids to determine whether these balanced fluids impact the immune response that occurs in sepsis. The Seplyte solutions were designed specifically for resuscitation in sepsis, and their composition is based upon three principles. First, it is known that acute kidney injury is a common organ dysfunction associated with sepsis so patients may have impaired renal K^+^ clearance [28], and hyperkalemia is a limiting factor affecting the utility of currently available balanced solutions (such as Ringer’s lactate and Plasma-Lyte) during large-volume resuscitation. With this in mind, the Seplyte solutions were made free of K^+^. Second, hyperlactatemia [29] and reduced lactate clearance [30, 31] have been associated with increased mortality in septic patients. Accordingly, the Seplyte solutions are buffered with acetate and gluconate, rather than lactate, to minimize the lactate load during organ dysfunction, while maintaining pH buffering and energy production capabilities through metabolism of acetate into bicarbonate [32] and metabolism of gluconate to produce ATP [33], respectively. This rationale is also being used by other investigators to study the use of Plasma-Lyte as a resuscitation fluid in sepsis (NCT02614040 and ANZICS CTG 1415-03). Lastly, these fluids were developed to satisfy the criteria of a balanced solution and thus contain chloride concentrations of 103 mM, which are similar to the concentrations found in plasma in order to minimize the risk for hyperchloremic acidosis [34, 35].

Upon testing, we found that the Seplyte resuscitation fluids are more beneficial than normal saline for decreasing leukocyte recruitment in the hepatic microvasculature during early sepsis in mice. Septic mice resuscitated with these novel balanced fluids exhibit leukocyte recruitment patterns similar to those seen in mice resuscitated with Ringer’s lactate. The cytokine profile observed for Seplyte H was also similar to Ringer’s lactate, with the exception of increased TNF-α in response to the novel fluids. These findings suggest that Seplyte H may be a suitable crystalloid fluid for resuscitation in sepsis, though follow-up studies would be required for confirmation. In contrast, Seplyte L elevated hepatic concentrations of most cytokines, which could potentially worsen the systemic inflammation that is characteristic of early sepsis. During such inflammation, there are increases in systemic neutrophil recruitment [36], which we observed through significantly reduced circulating neutrophil counts in most septic animals, presumably due to increased migration out of the circulation and into infected tissues. However, Seplyte L did not dramatically reduce circulating neutrophil counts compared to other fluids; therefore, it is unlikely that the increases in hepatic cytokines exacerbated the systemic immune response in our model. It is interesting that Seplyte L decreased leukocyte recruitment in the liver despite elevating hepatic pro-inflammatory cytokines. We speculate that simultaneous increases in anti-inflammatory cytokines could explain this discrepancy as the observed increases in hepatic IL-10 support this hypothesis, although more anti-inflammatory cytokines would need to be evaluated for confirmation.

Whether or not elevated TNF-α is detrimental in sepsis remains controversial. Some studies show that circulating TNF-α may contribute to hepatocellular dysfunction in early sepsis [37] and that TNF-α concentrations in plasma are a predictor of mortality in septic mice [38]. On the other hand, TNF-α blockade using antibodies failed to protect mice undergoing CLP [39, 40], meanwhile protection was observed following recombinant human TNF-α injections in septic rats [41]. Importantly, we measured hepatic TNF-α in this study, rather than plasma concentrations, due to the greater relevance of localized cytokine concentrations for leukocyte recruitment in the liver. Indeed, Andrejko and Deutschman found that hepatic, but not circulating, TNF-α modified gene expression in the liver via paracrine function [42], which suggests that measuring TNF-α in plasma is not sufficient to determine the concentrations and effects in the liver. It is probable that a balance, with moderately elevated TNF-α in the tissues while inhibiting excess TNF-α in the circulation, would maintain efficient leukocyte recruitment to sites of infection while limiting harmful systemic effects, such as excessive systemic leukocyte recruitment and microvascular leakage [43]. If this hypothesis is true, then the hepatic increases in TNF-α observed in response to resuscitation with our novel fluids may actually be protective, although additional studies would be necessary to make such conclusions. Future studies should consider comparing the effective concentrations of systemic and local cytokines, in an attempt to reconcile the literature about the importance and detriment of TNF-α in early sepsis.

In this study, we have shown that fluid resuscitation with albumin has minimal effects on hepatic leukocyte recruitment in a murine model of early sepsis. These results contrast with previous findings that albumin fluid resuscitation can attenuate leukocyte-endothelial cell interactions in vitro [44] and in vivo [45], in models of septic and hemorrhagic shock, respectively. Supplementing crystalloid fluids with albumin also did not alter circulating neutrophil, monocyte, or lymphocyte counts, which suggests that systemic leukocyte recruitment is unaffected by albumin, and is consistent with the data from our intravital microscopy experiments demonstrating minimal effects of albumin on hepatic leukocyte recruitment. Furthermore, albumin did not affect the concentrations of most hepatic cytokines measured in this study, further supporting that resuscitating with albumin does not have a significant effect on hepatic inflammation during early sepsis. Resuscitation with albumin also did not improve sinusoidal blood flow in this study, which contrasts with our previous findings that other colloids, namely starches [21] and alpha-1 acid glycoprotein [22], help to restore flow to nearly normal levels in septic mice. This differential activity between albumin and other colloids further highlights the need to extensively study the physiological effects of colloids in fluid resuscitation prior to clinical use.

In this study, we also measured cfDNA as a sepsis biomarker [25]. The release of cfDNA from neutrophils during sepsis has been identified as a protective mechanism to trap pathogens in so-called neutrophil extracellular traps (NETs) [46]. However, NETs can also activate endothelial cells and induce collateral tissue damage in the lungs, heart, and liver [47, 48]. Studies investigating the effects of recombinant human DNase on the effects of NETs in murine models of sepsis have demonstrated benefits from delayed DNase treatment [49] and from DNase as an adjunctive treatment to antibiotics [48]. Another source of cfDNA in sepsis is mitochondrial DNA [50], which was found to contribute to acute kidney injury and cytokine production in polymicrobial sepsis models [51]. Our results demonstrate that the type of fluid used for resuscitation does not affect plasma cfDNA concentrations during early sepsis in mice. Furthermore, the presence or absence of albumin in these resuscitation fluids did not appear to modulate cfDNA concentrations during early sepsis. These findings suggest that the choice of resuscitation fluid is unlikely to affect production of NETs during early sepsis and that cfDNA may be a reasonable biomarker to confirm the induction of sepsis in mice undergoing CLP.

There are some limitations to our pilot study comparing traditional and novel resuscitation fluids in early sepsis. We focused on an early time point in order to compare data with our previous microscopy studies of the liver microcirculation during early sepsis. Future studies should extend the time point to gain insight into the effects of fluid resuscitation on leukocyte recruitment, inflammation, and organ injury during later stages of sepsis. Our study is also limited by its focus on the liver; thus, investigating the effects of these resuscitation fluids on other organs that become dysfunctional during sepsis, such as the lungs and kidneys, would be an important avenue for future work.

## Conclusions

The novel balanced crystalloid solution of Seplyte H provides some anti-inflammatory benefit over normal saline by reducing leukocyte recruitment in the hepatic microcirculation with minimal effects on the hepatic cytokine profile, although future studies are needed to determine whether the increases in hepatic TNF-α are detrimental. Supplementing crystalloid fluids with albumin has only modest effects on leukocyte recruitment in the liver microcirculation and does not appear to alter hepatic cytokine production or circulating leukocyte counts relative to the crystalloids alone. Finally, plasma cfDNA is unaffected by the choice of resuscitation fluid and thus may be a useful biomarker in experimental studies of fluid resuscitation during sepsis.

## Additional file

Additional file 1: Digital supplemental content. (DOCX 3132 kb)

## Abbreviations

ANOVA: Analysis of variance
cfDNA: Cell-free DNA
CLP: Cecal ligation and puncture
IL: Interleukin
KC/CXCL1: Chemokine (C-X-C motif) ligand 1
LPS: Lipopolysaccharide
MCP-1/CCL2: Monocyte chemoattractant protein-1
NET: Neutrophil extracellular trap
SEM: Standard error of the mean
TNF-α: Tumor necrosis factor-alpha
