# Supplementary material for: Resuscitation fluid composition affects hepatic inflammation in a murine model of early sepsis
Source: Intensive Care Med Exp. 2017 Jan 19;5:5. doi: 10.1186/s40635-017-0118-5 (PMC5247397; doi:10.1186/s40635-017-0118-5)
Supplement: Additional file 1: — Digital supplemental content. (DOCX 3132 kb) [file 40635_2017_118_MOESM1_ESM.docx]

Digital Supplemental Content

***Methods:***

***Buffered electrolyte solutions in naïve and endotoxin-treated mice***

C57Bl/6 mice were used to evaluate the effects of the novel crystalloid solutions on leukocyte recruitment in the hepatic microvasculature and on plasma cfDNA levels. At time = 0 h, four groups of mice (n=6) were given intraperitoneal injections of 5 mg/kg lipopolysaccharide (LPS) from *Escherichia Coli* (Sigma, Oakville, ON), and control groups (n=6) were given an equal volume of normal saline. After 3.75 h, a catheter was inserted into the right internal jugular vein, through which mice were given an intravenous bolus of 0.5 mL (20 mL/kg) of either normal saline, Ringer’s lactate, Seplyte L or Seplyte H. Intravital microscopy (see Methods) was performed to visualize the liver microcirculation at 4 h after initial treatment with LPS or control. Vessels in the hepatic microvasculature were recorded at 0, 5, 10, 15 and 20 min after exteriorization of the liver, for 2 min at each time point to obtain an average number of cells per minute per field of view. Blood was collected and plasma was stored immediately following intravital microscopy for subsequent measurement of cfDNA.

**Results:**

Hepatic leukocyte recruitment in response to control and novel crystalloids in naïve and endotoxemic mice

To determine the microcirculatory response to our novel crystalloid solutions, healthy naïve mice were compared to *E. coli* LPS (5 mg/kg) treated mice given the same fluids (normal saline, Ringer’s Lactate, Seplyte L or Seplyte H). Leukocyte rolling flux in the hepatic post-sinusoidal venules was not significantly different between naïve and LPS-treated mice in all fluid treatment groups. Also, no significant difference in hepatic leukocyte rolling was found between control solutions (normal saline and Ringer’s lactate) and the novel Seplyte solutions for either naïve or LPS-treated mice (Figure S1). In naïve mice, leukocyte adhesion in the post-sinusoidal venules was not significantly different between the novel and control fluids (Figure S2). LPS treatment significantly increased levels of post-sinusoidal adhesion for all fluid groups compared to naïve controls. Furthermore, LPS-treated mice given either Ringer’s lactate or Seplyte L had significantly higher post-sinusoidal adhesion than LPS-treated mice given Seplyte H (Figure S2). Similarly, sinusoidal leukocyte adhesion was significantly higher in LPS-treated mice for each fluid regimen; however, treatment with novel Seplyte solutions showed no significant effect on sinusoidal adhesion compared to control fluids (Figure S3). These findings suggest that the novel Seplyte solutions do not acutely stimulate leukocyte recruitment in the hepatic microvasculature.

Seplyte L and H do not elevate cfDNA levels in endotoxemic mice

Levels of cfDNA were determined in the plasma of naïve and LPS-stimulated mice at 4 hours. LPS endotoxemia significantly increased the cfDNA levels in mice given normal saline, but did not significantly increase cfDNA levels in mice given Seplyte L and Seplyte H (Figure S4). These findings suggest that the novel Seplyte solutions do not acutely stimulate release of cfDNA from neutrophils or damaged tissue.

**DSC Figure S1**. Leukocyte rolling in hepatic post-sinusoidal venules of C57Bl/6 mice at 4 hours after LPS treatment. *n*=5 all groups. There were no significant differences between comparable groups. LPS, lipopolysaccharide.

**DSC Figure S2**. Leukocyte adhesion in hepatic post-sinusoidal venules of C57Bl/6 mice at 4 hours after LPS treatment. *n*=5 all groups. * *p*<0.05 compared with naïve, ^ *p*<0.05 compared with LPS normal saline, ~ *p*<0.05 compared with LPS Seplyte H. LPS, lipopolysaccharide.


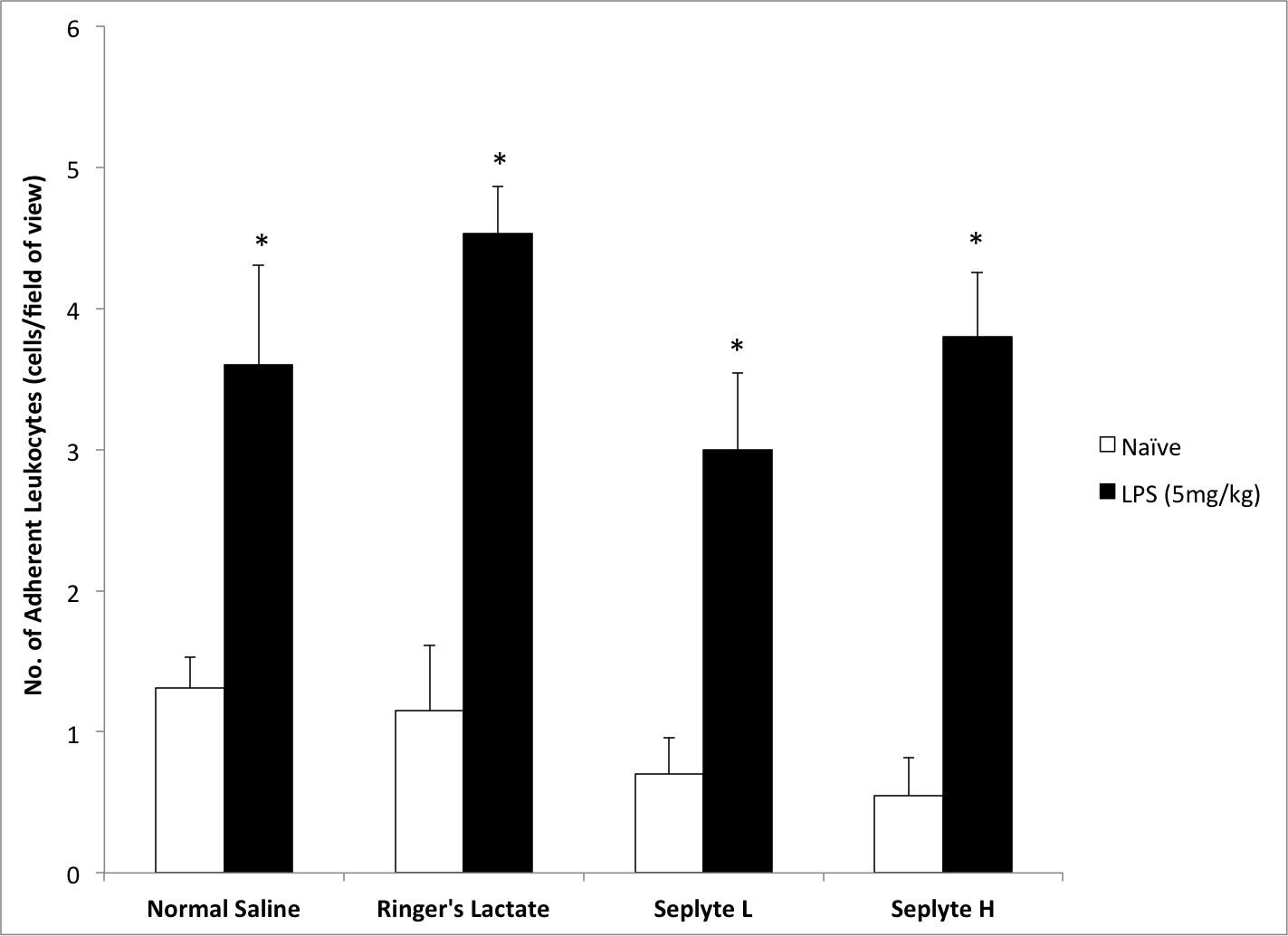


**DSC Figure S3**. Leukocyte adhesion in hepatic sinusoids of C57Bl/6 mice at 4 hours after LPS treatment. *n*=5 all groups. * *p*<0.05 compared with naïve. LPS, lipopolysaccharide.


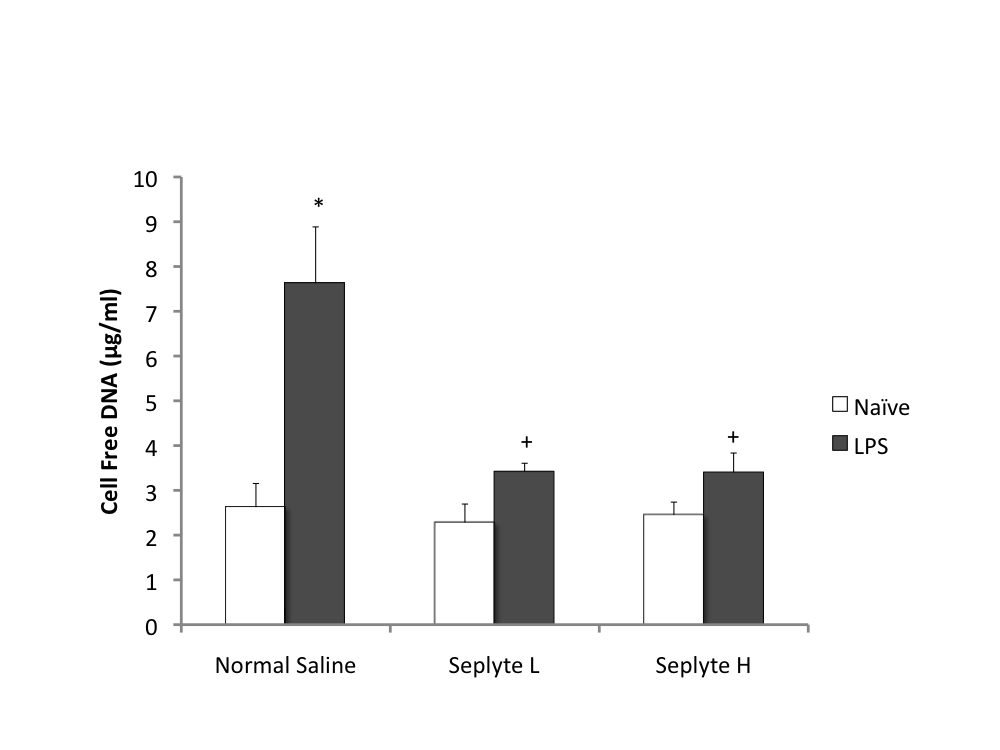


**DSC Figure S4.** Cell free DNA isolated from plasma of naïve and LPS-treated C57Bl/6 mice at 4 h. * p < 0.05 compared with naïve, + p < 0.05 compared with LPS normal saline. LPS, lipopolysaccharide.
